# CTEC: a cross-tabulation ensemble clustering approach for single-cell RNA sequencing data analysis

**DOI:** 10.1093/bioinformatics/btae130

**Published:** 2024-03-29

**Authors:** Liang Wang, Chenyang Hong, Jiangning Song, Jianhua Yao

**Affiliations:** AI Lab, Shenzhen 518054, China; Department of Computer Science and Engineering, The Chinese University of Hong Kong, Hong Kong, 999077, China; Biomedicine Discovery Institute and Department of Biochemistry and Molecular Biology, Monash University, Clayton, VIC 3800, Australia; AI Lab, Shenzhen 518054, China

## Abstract

**Motivation:**

Cell-type clustering is a crucial first step for single-cell RNA-seq data analysis. However, existing clustering methods often provide different results on cluster assignments with respect to their own data pre-processing, choice of distance metrics, and strategies of feature extraction, thereby limiting their practical applications.

**Results:**

We propose Cross-Tabulation Ensemble Clustering (CTEC) method that formulates two re-clustering strategies (distribution- and outlier-based) via cross-tabulation. Benchmarking experiments on five scRNA-Seq datasets illustrate that the proposed CTEC method offers significant improvements over the individual clustering methods. Moreover, CTEC-DB outperforms the state-of-the-art ensemble methods for single-cell data clustering, with 45.4% and 17.1% improvement over the single-cell aggregated from ensemble clustering method (SAFE) and the single-cell aggregated clustering via Mixture model ensemble method (SAME), respectively, on the two-method ensemble test.

**Availability and implementation:**

The source code of the benchmark in this work is available at the GitHub repository https://github.com/LWCHN/CTEC.git.

## 1 Introduction

Single-cell RNA sequencing (scRNA-Seq) technologies enable researchers to better characterize and understand cellular heterogeneity and cell types. Accordingly, tools for scRNA-seq data analysis are essential for cell-type composition understanding (Grün *et al.* 2015, Jia *et al.* 2017). As a basic step, single-cell clustering plays an important role in downstream analysis such as cell annotation, single-cell differential expression, and single-cell trajectory analysis (Zhu *et al.* 2018). To date, a variety of methods have been developed for clustering analysis of scRNA-Seq data, such as Louvain in Seurat (Satija *et al.* 2015), Dirichlet mixture model based method (DIMM-SC) (Sun *et al.* 2018), DendroSplit (Zhang *et al.* 2018), Leiden and Louvain in Single-Cell Analysis in Python (Scanpy) (Wolf *et al.* 2018) and Spectral-based method in Pegasus (Li *et al.* 2020a), etc. However, most of these existing algorithms only work well under certain conditions and do not generalize to different data distributions. The challenges for scRNA-seq clustering are as follows: Firstly, for a large number of scRNA-seq data there is no prior knowledge of cell type composition (Yang *et al.* 2019). Secondly, biological domain knowledge may be required to assess the clustering results. Moreover, the same parameters may not be optimal for different scRNA-seq datasets. Considering that each individual clustering algorithm may address part of such challenges, we propose an ensemble approach to create a better clustering solution.

A few ensemble methods have been previously proposed for integrating the clustering solutions. For example, the method of Single-Cell Consensus Clustering (SC3) generated different sets of clustering results by evaluating the subsets of parameter space (Kiselev *et al.* 2017). A consensus matrix was then calculated using a cluster-based similarity partitioning algorithm. It summarized the probability of each pair of cells that were predicted in the same cluster, and all different clusters were subsequently combined according to the probability scores. The Single-cell Aggregated (From Ensemble) clustering (SAFE) method (Yang *et al.* 2019) embedded the results of four individual single-cell clustering methods by hypergraph and then applied one of the hypergraph-based partitioning algorithms discussed in Strehl and Ghosh (2003). This work optimized both the cluster number and the cluster assignment. The four selected methods were generally developed based on principal component analysis (PCA), community detection, and k-means clustering techniques. However, the features from individual cell may not be well considered during the process. The Single-cell Aggregated clustering via Mixture model Ensemble (SAME) clustering method (Huh *et al.* 2020) was a follow-up to the SAFE method, which built an ensemble solution by the expectation-maximization (EM) algorithm, assuming that the individual clustering labels are drawn from a mixture of multivariate multinomial distributions. Although the comparisons to the SAFE method showed improvements on most datasets, the time-consuming step made it difficult to scale up and apply on large datasets since the SAME method internally running multiple time of the ensemble method for determining a converged final estimated number of clusters.

In this study, we develop a new method for scRNA-seq data analysis, termed single-cell Cross-Tabulation Ensemble Clustering (CTEC). We first show that the proposed method can integrate a pair of clustering results by effectively taking advantage of two state-of-the-art methods, the community detection-based Leiden method (Traag *et al.* 2019) and the unsupervised Deep Embedding algorithm that clusters Single-Cell RNA-seq data (DESC) (Li *et al.* 2020b) by iteratively optimizing a clustering objective function. A cross-tabulation of the clustering results from the two individual methods is generated. After that, one of two correction schemes is carried out under different circumstances: outlier-based clustering correction and distribution-based clustering correction. Accordingly, the ensembled clustering results of the two individual methods are iteratively modified from the cross-tabulation analysis until a stable clustering result is obtained from one of the individual methods. Further, we propose a progressive scheme to iteratively ensemble multiple methods via pair-wise cross-tabulation. We show that the CTEC method provides a satisfactory clustering ensemble without prior knowledge of cell types. Moreover, CTEC also shows robustness when using the same default parameters of each individual method to process different large-scale scRNA-Seq datasets in our experiments.

## 2 Materials and methods

### 2.1 Overview of CTEC

**Figure 1.**
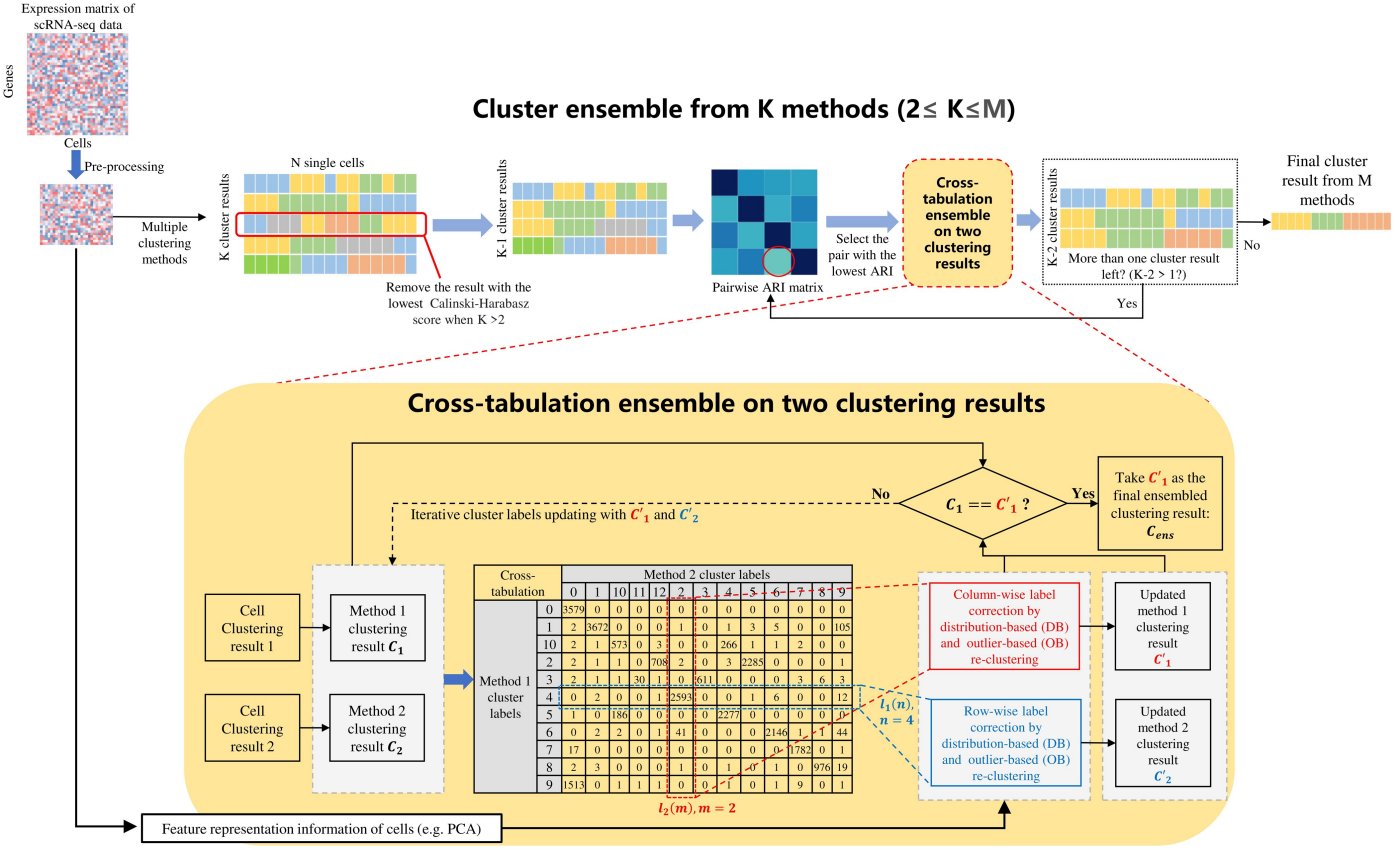
An overview of the architecture of the proposed CTEC method. A Cross-tabulation ensemble operating on two clustering results is used as a module for the multi-method ensemble based on the cluster quality evaluation and pairwise cross-tabulation rectification.

Figure 1 illustrates an overview of the architecture of our proposed CTEC method in two main parts. Provided with two clustering algorithms in the first part of the cross-tabulation ensemble, CTEC aims to take advantage of each method using cross-tabulation rectification, eventually correcting one method from another all the way to the final consensus clustering results. In the second part of the cluster ensemble from K methods, we propose a strategy to ensemble the multiple clustering results by using the first part as one module.

### 2.2 Benchmarking datasets

We collected five benchmarking datasets that have been commonly used for performance evaluation of recent works in the literature (Li *et al.* 2020b, Lakkis *et al.* 2021). They are available by the accession numbers, which included: (i) Macaque: Bipolar cells from macaque retina (GSE118480); (ii) PBMC: human peripheral blood mononuclear cell (PBMC) data (GSE96583); (iii) Cortex: mouse cortex data (Single Cell Portal SCP425); (iv) Pancreas: human pancreatic islet data: CelSeq (GSE81076), CelSeq2 (GSE85241), Fluidigm C1 (GSE86469), and SMART-Seq2 (E-MTAB-5061); (v) Paul15: myeloid progenitor subpopulations from the mouse bone marrow data by Paul in 2015 (GSE727857) A statistical summary table of these datasets is described in the Supplementary.

### 2.3 Ensemble between two clustering results

#### 2.3.1 Cross-tabulation generation and label matching

The cross-tabulation builds upon a 2D table that summarizes the relationship between two individual clustering results (Barghoorn 1996). We first construct a pivot table from the number of cells corresponding to each cluster of different methods, where row and column variables represent the clustering labels of each method respectively. It is noted that different labels in two clustering methods may represent the same cluster. Hence, in order to combine the clustering results from different methods, a cluster labels correspondence must be established between the two methods. Inspired by the idea of cluster label matching in Wang and Chen (2020), we specify the two individual methods as the row (method 1) and column (method 2) of the cross table, respectively. From the cross-tabulation in Fig. 1, we have one label $l_{2}(m)$ of method 2, and *m* is the cluster label number. The row index of the largest number in the corresponding column of $l_{2}(m)$ indicates the corresponding label $l_{1}(n)$ from method 1, which forms a pair of corresponding labels (i.e. $l_{2}(m),\;l_{1}(n)$) Wang and Chen (2020). For each cluster of the column, label instances are mainly divided into two groups: matched cells and unmatched cells. Matched cells are those cell samples definitely belonging to the pair of corresponding labels, i.e. $l_{2}(m)$and $l_{1}(n)$. Unmatched cells are those cell samples belonging to other clusters of method 1 except cluster $l_{1}(n)$.

#### 2.3.2 Distribution-based and outlier-based re-clustering strategies

Re-clustering is a step to examine the cluster label of each cell from the input methods and relabel them according to the cross table. For better clarification of re-clustering, we take the column-wise analysis as an example. We consider all the cells in a single cluster $l_{2}(m)$ of method 2, which is one column of the cross-tabulation. All these cells may belong to several clusters in method 1. They are named ‘matched cell’ if they belong to the matched labels, i.e. the row cluster $l_{1}(n)$ and the column cluster $l_{2}(m)$. The other cells are named ‘unmatched cells,’ whose labels are examined to determine whether they should be re-labeled to $l_{1}(n)$ as the matched cells. Distribution-based re-clustering (DB) and outlier-based re-clustering (OB) are developed for unmatched cell re-clustering. Two different clustering results will be obtained based on the choice of distribution- or outlier-based re-clustering, which are discussed in detail in the Results section.

In the distribution-based re-clustering scheme, for each label of method 1, the coefficient of variation (CV) of the distribution of the number of cells among the clusters in method 2 is calculated. The higher CV value indicates that the unmatched cells might be inappropriately clustered by method 1 and they should be re-clustered with the label $l_{1}(n)$. A threshold *CV_th_* can be adopted to decide the re-clustering operation. For a column *m* in the cross-tabulation with the label $l_{2}(m)$ of the method 2, *CV*(*m*) can be calculated as follows and unmatched cells are accordingly re-clustered:

(1) $$CV(m)=\frac{\mathrm{STD}(m)}{\mathrm{MEAN}(m)},$$

(2) $$l_{1}(i)=\{\begin{array}{c} \begin{array}{l} l_{1}(n),\;if\;CV(m)<CV_{th}\\ \mathrm{keep}\;\mathrm{the}\;\mathrm{label}\;l_{1}(i),\;if\;CV(m)\geq CV_{th} \end{array} \end{array},$$

where *STD* represents the standard deviation and *MEAN* represents the mean value of the cell quantity of one cluster, respectively. Hence, all the unmatched cells in the column *m* of the cross-tabulation are re-considered according to the threshold *CV_th_*. In addition, the value of *CV_th_* is initialized as 2 and is reduced iteratively at the rate of 0.2 until it reaches 0. The iteration will stop early when no more cell labels are updated between the two consecutive *CV_th_* values.

The distribution-based re-clustering works under the assumption that higher CV indicates the clusters of matched cells are much more reliable than those of the unmatched cells. Hence, all the unmatched cells in a sub-group are re-clustered altogether. As a complementary solution, we develop the outlier-based re-clustering strategy by taking into account the feature representation of each cell. For the outlier-based re-clustering, we apply the outlier detection method instead of the CV analysis in the distribution-based re-clustering. The features of each cell in the single cluster $l_{2}(m)$ of method 2 are evaluated.

For a better understanding, we consider that all the cells in a single cluster $l_{2}(m)$ of method 2 as a subgroup (i.e. one column of the cross-tabulation). Then, we implement a subgroup clustering with two sources of prior knowledge: (i) the subgroup is needed to be re-clustered into two new parts, one of which is the new matched-cell part and another is the new unmatched-cell part; (ii) ideally, the new matched-cell part covers most of this subgroup while the new unmatched-cell part is considered as the outlier cell samples of the subgroup. To detect the outlier cells, the COPula-based Outlier Detector (COPOD) (Li *et al.* 2020c) method was adopted and the principal component analysis (PCA) coordinates of scRNA-seq expression matrix for each cell sample are obtained by Scanpy (version 1.8.2 with Python version 3.8.0) and used as the input to the COPOD method. COPOD is a parameter-free outlier detection algorithm. It uses copula models for the first time for explainable outlier detection tasks, and provides a comparable capability in terms of the detection accuracy and computational cost.

Similarly, using the same cross-tabulation, a row-wise label correction is processed by choosing the distribution-based re-clustering or outlier-based re-clustering as discussed above. Hence, we obtain the updated clustering result $C_{1}^{\prime }$ for the row and updated clustering result $C_{2}^{\prime }$ for the column, respectively. To perform the label re-clustering iteratively, we compare $C_{1}^{\prime }$ with the input clustering result (method 1) for cross-tabulation. If they are not the same, then $C_{1}^{\prime }$ and $C_{2}^{\prime }$ will be used as the input for another round of label re-clustering; otherwise, $C_{1}^{\prime }$ is considered as the final ensemble clustering result and named *C_ens_*. Therefore, the number of CTEC clusters is based on that of clusters of the input ensembled methods. More cross-table iterations may reduce the CTEC clusters number gradually and converge to a stable cluster number.

#### 2.3.3 Ensemble clustering using two state-of-the-art methods

We first demonstrate the power of CTEC by integrating two state-of-the-art single-cell clustering methods, namely Leiden (Traag *et al.* 2019) and DESC (Li *et al.* 2020b). These two methods generate clustering results that are complementary with each other, which makes it suitable for applying our ensemble method to further improve the clustering results.

The Leiden method optimizes the results of incorrectly connected communities from the Louvain algorithm (Blondel *et al.* 2008). Using this method, the subsets of all communities can be locally and optimally assigned and converge to a partition. The Leiden method is implemented using Scanpy (Wolf *et al.* 2018), which is a scalable toolkit for analyzing single-cell gene expression data. Scanpy provides a standard workflow of scRNA-seq analysis in Python. As the granularity of the clustering results is sensitive to the ‘resolution’ parameter that controls the size of clusters of the Leiden method, we perform a parameter optimization for a fine clustering result (Stacey *et al.* 2021). To obtain clustering results with Leiden, we first compute the PCA using the *scanpy.tl.pca* function with *svd_solver=‘arpack’*. Then, we compute the neighborhood graph using the *scanpy.pp.neighbors* function with n_neighbors=10. Finally, we use all the default parameters of the function *scanpy.tl.leiden* described at official link https://scanpy.readthedocs.io, e.g. with the default *resolution *=* *1.0.

DESC is a deep learning-based framework for scRNA-seq clustering. It iteratively optimizes a clustering objective function to achieve an unsupervised deep embedding self-learning, which benefits batch effects correction. In this method, the low-dimensional representation of the scRNA-seq expression matrix is learned from a stacked auto-encoder. Then the Louvain algorithm is applied to the low-dimensional feature representation data of cell samples for an initial clustering with the given number of true clusters. The final clusters are refined iteratively based on KL divergence as described in Xie *et al.* (2016). As a soft clustering method, DESC proposes the probabilities of cluster assignment to facilitate the biological interpretation of discrete and pseudo-temporal structure of cells. It can be observed that DESC generates a satisfying clustering result when the ‘resolution’ parameter for the Louvain algorithm is optimized for the initial clustering. For DESC method, we use all the default parameters described on its original open source at link https://github.com/eleozzr/desc. For example, *resolution *=* *1.0. In addition, batch size was set to 256 or 1024 for the dataset with fewer or more than 10 000 cells.

#### 2.3.4 Multiple methods ensemble

We have introduced the CTEC method for ensemble clustering from two individual methods. For a general purpose, more than two clustering methods (or one method with multiple parameter settings) can be ensembled to obtain more robust clustering results, since different methods may provide complementary information to each other. Multiple clustering-based ensemble has been proposed in SAFE-clustering (Yang *et al.* 2019) and SAME-clustering (Huh *et al.* 2020). Here, we propose an approach to ensemble multiple methods based on the pairwise cross-tabulation rectification. The workflow is shown in the module of cluster ensemble from *K* methods in Fig. 1. Assume we have *N* individual clustering results that need to be ensembled, a cluster quality evaluation is applied based on the unsupervised Calinski-Harabasz (CH) score (Caliński and Harabasz 1974), which is a metric for clustering evaluation without the ground truth labels and is highly correlated with the ground-truth ARI value (Supplementary Fig. S11). For the cluster quality evaluation, the worst individual clustering result according to the CH score is first removed in order to alleviate the effect of potential bad clustering result on the final ensemble result. Then, for the remaining *N—*1 individual clustering results, the pairwise adjusted random index (ARI) scores are calculated, which are used to evaluate the consistency between different clustering results. The pair of clustering results with the lowest ARI score (most dissimilar) will be selected and used to perform the two-methods cross-tabulation re-clustering to get the consensus clustering result. Next, the pair of clustering results will be removed from the queue and the newly merged clustering result will be added. Accordingly, the number of clustering results that need to be integrated will decrease by one. If there are still more than one clustering results, the process will continue from calculating the pairwise ARI scores until there is only one clustering result left, which is the output clustering ensemble from *K* methods.

For the following benchmarking experiments, we use five individual methods (Huh *et al.* 2020) including SC3 (Kiselev *et al.* 2017), CIDR (Lin *et al.* 2017), Seurat (default Louvain algorithm) (Butler *et al.* 2018), t-SNE+*k*-means (Van der Maaten and Hinton 2008) and SIMLR (Van der Maaten and Hinton 2008, Wang *et al.* 2017) to obtain five sets of clustering solutions. These methods were applied in the SAME-clustering works (Huh *et al.* 2020), which enable us to make a fair final comparison between different ensemble methods. Particularly, the method t-SNE + *k*-means is named as Kmeans for short in this work. For the five individual clustering methods, we follow the same parameter settings as in the SAME-clustering paper (Huh *et al.* 2020). For the expected number of clusters in the SC3 and SIMLR methods, the average number of clusters in the results of the other three methods is used instead of using the cluster number estimation function provided by the methods, which may lead to a very large number of clusters for some datasets. We also generate two sets of results for the SAFE and SAME ensemble methods with all the five or four (by removing the one with the lowest CH score) clustering methods as the input, and the resulting ARI values are similar for both input settings (For details refer to the Supplementary Section: Ensemble with SAFE and SAME method with four or five input methods).

### 2.4 Evaluation metrics for clustering

The true cell type labels of all our benchmarking datasets have been pre-defined by the original papers, which are used as the gold standard. The clustering performance is mainly evaluated using two metrics: ARI and normalized mutual information (NMI) (Gates and Ahn 2017). The larger values of ARI and NMI represent better clustering accuracy. They are respectively calculated as follows:

(3) $$\mathrm{ARI}=\frac{\sum_{pt}\left(\begin{array}{c} n_{pt}\\ 2 \end{array}\right)-\left[\sum_{p}\left(\begin{array}{c} a_{p}\\ 2 \end{array}\right)\sum_{t}\left(\begin{array}{c} b_{t}\\ 2 \end{array}\right)\right]/\left(\begin{array}{c} n\\ 2 \end{array}\right)}{\frac{1}{2}\left[\sum_{p}\left(\begin{array}{c} a_{p}\\ 2 \end{array}\right)+\sum_{t}\left(\begin{array}{c} b_{t}\\ 2 \end{array}\right)\right]\;-\;\left[\sum_{p}\left(\begin{array}{c} a_{p}\\ 2 \end{array}\right)\sum_{t}\left(\begin{array}{c} b_{t}\\ 2 \end{array}\right)\right]/\left(\begin{array}{c} n\\ 2 \end{array}\right)}$$

(4) $$\mathrm{NMI}=2\times \frac{\sum_{pt}\frac{n_{pt}}{n}\;\text{log}\;\left(\frac{n\times n_{pt}}{a_{p}\times b_{t}}\right)}{\sum_{p}\frac{a_{p}}{n}\;\text{log}\;\left(\frac{n}{a_{p}}\right)+\sum_{t}\frac{b_{t}}{n}\;\text{log}\;\left(\frac{n}{b_{t}}\right)}$$

where *n* is the total number of cells, *n_pt_* is the number of cells in both cluster *p* of the predicted cluster and cluster t of the gold standard cell type annotation. *a_p_* is the total number of cells in the cluster *p* of the predicted cluster, *b_t_* is the total number of cells in the cluster *t* of the gold standard cell type annotation, respectively.

## 3 Results

Our experiments were conducted on the five benchmarking datasets that represent a wide variety of species, sequencing techniques and numbers of cell types. All individual clustering methods used the same default parameters. We conducted the similar preprocessing on the five benchmarking datasets. Firstly, we logarithmized the data matrix by ‘scanpy.pp.log1p,’ then selected the 1000 highly variable genes by ‘scanpy.pp.highly_variable_genes,’ and finally, we used, the batch correction and scale by ‘scanpy.pp.combat’ and ‘scanpy.pp.scale’ with the parameter max_value=10. For the batch correction, we used the ‘macaque_id,’ ‘stim,’ ‘CellType,’ and ‘protocol’ for the datasets Macaque, PBMC, Cortex, and Pancreas, respectively. For the methods of SAME, SC3, CIDR, Seurat, Kmeans, and SIMLR, they were implemented based on the default parameters as described in previous study (Yang *et al.* 2019) on the original open source link. For the SAFE method, we used all the default parameters described on its original open source link (Huh *et al.* 2020). For the proposed CTEC method, we evaluated different setups with different combination of individual clustering methods and distribution-based/outlier-based re-clustering strategies.

### 3.1 Ensemble of two state-of-the-art methods

**Figure 2.**
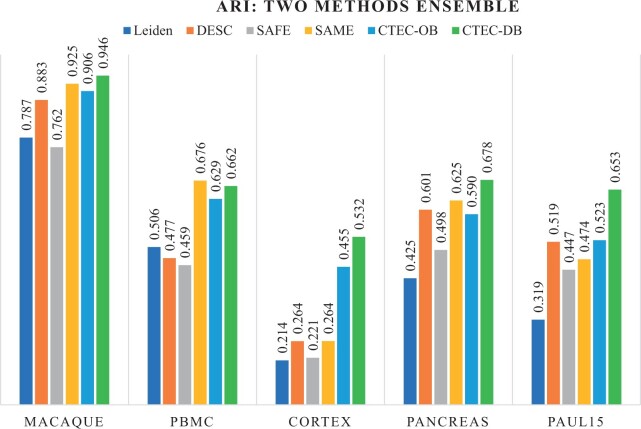
Performance evaluation of the SAFE, SAME, and CTEC methods based on the ensemble of two methods (Leiden and DESC) in terms of ARI on the benchmark datasets.

First, we evaluated the ensemble of two state-of-the-art individual clustering methods, i.e. Leiden and DESC, compared to the ensemble results produced by the SAFE-clustering and SAME-clustering methods. The ARI values of the clustering results produced by the individual methods as well as their consensus clustering results from different ensemble methods are shown in Fig. 2, while the corresponding NMI values can be found in the Supplementary Fig. S5. From the comparison of ARI across five datasets, it can be observed that both of our CTEC with the outlier-based re-clustering (CTEC-OB) and CTEC with distribution-based re-clustering (CTEC-DB) methods outperformed the individual Leiden and DESC methods with the only exception for the smallest dataset (Paul15), for which the resulting ARI of our ensemble methods was the second best compared to the two individual methods (closer to the DESC result), since the number of cells per each cluster was too small to provide confident support for the cross-tabulation step. As for the other two ensemble methods, CTEC produced better clustering performance than the SAFE-clustering method on all five datasets, and it also outperformed the SAME-clustering on three datasets while achieving comparable results on the other two datasets. In terms of ARI, our CTEC-OB and CTEC-DB methods improved the performance from 38.7% to 54.2% compared with Leiden method, and from 13.1% to 26.5% with DESC methods, respectively. In terms of the ensemble methods, CTEC-OB improved the ARI value of the clustering results by 16.5% (compared to the SAFE-clustering) and 32.2% (compared to the SAME-clustering) on average across the five datasets, while CTEC-DB improved the ARI of the clustering results by 19.4% (compared to the SAFE-clustering) and 35.5% (compared to the SAME-clustering) on average.

#### 3.1.1 Application to the cortex dataset

**Figure 3.**
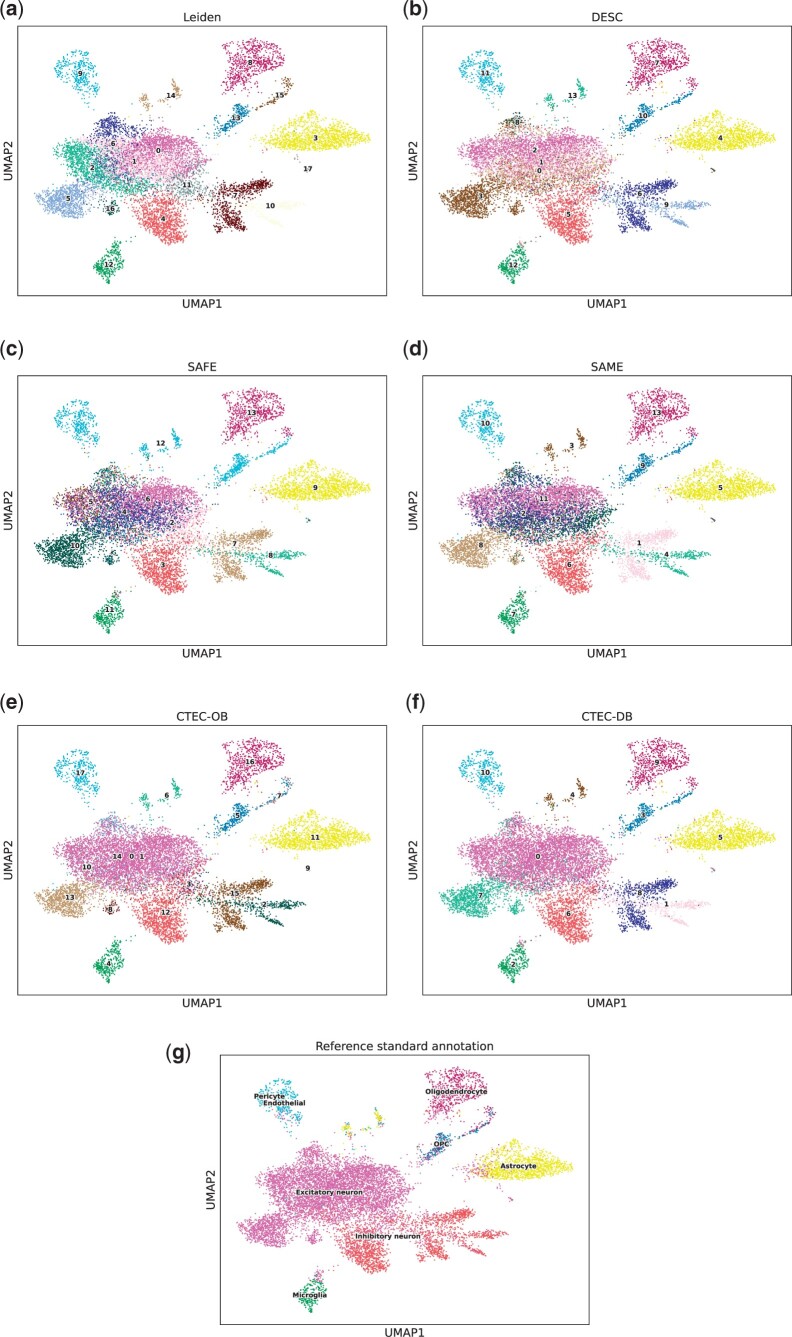
UMAP plots of two methods ensembled on the Cortex dataset. (a) Leiden, (b) DESC, (c) SAFE, (d) SAME, (e) CTEC-OB, (f) CTEC-DB, (g) Reference standard annotation. Particularly, compared with SAFE and SAME, CTEC yields more accurate clustering results for excitatory neuron cells. In contrast, the clustering results are more diffused in Leiden, DESC, SAME, and SAME for excitatory neuron cells.

The cells of the Cortex dataset were generated using four different scRNA-seq protocols, which brings a great challenge for any single-cell clustering method due to the batch effect. In addition, the serious unbalanced quantity of cell types of this data makes it difficult for single-cell clustering of the rare cell (Lakkis *et al.* 2021). Due to these constraints, the Leiden and DESC methods only achieved low ARIs (< 0.26) and NMIs (< 0.55) using their default parameters. As for the two competing ensemble methods, the ARI (0.22) of the ensemble clustering produced by SAFE-clustering was slightly better than the original individual results, while the SAME-clustering achieved the ARI value of 0.26. However, the CTEC results provided an observable improvement of ARI (>0.45) and NMI (>0.57), and CTEC-DB performed the best in terms of both ARI (0.53) and NMI (0.62). Specifically, Fig. 3 shows uniform manifold approximation and projection (UMAP) (McInnes *et al.* 2018) plots of the methods for the Cortex dataset. We concluded that CTEC-DB correctly clustered most of the excitatory neuron cells (cluster #0 and cluster #7) while the excitatory neuron cells were predicted approximately by eight clusters in Leiden, by seven clusters in DESC (#0, #1, #2, #3, #8, #11, and #13) and by six clusters in SAFE (#1, #2, #4, #5, #6, and #10), four clusters in SAME clustering result (#2, #8, #11, and #12). Therefore, our proposed CTEC method demonstrated excellent capabilities of improving the low-quality individual clustering results produced by Leiden and DESC and generating ensemble clustering with high-quality. For the remaining datasets, the UMAP plots can be found in the Supplementary Section: Ensemble with two methods.

### 3.2 Ensemble of multiple clustering methods

**Figure 4.**
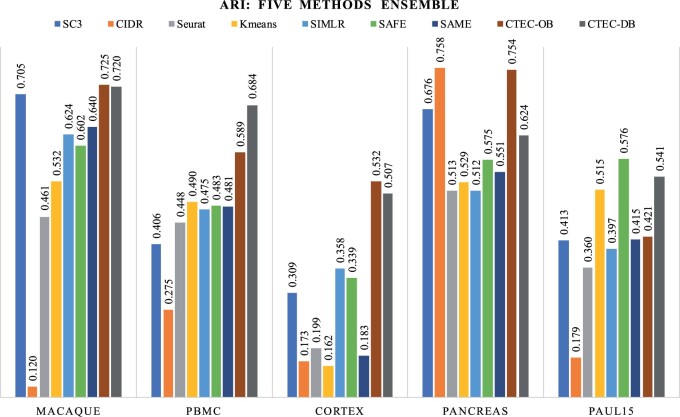
Performance evaluation of the five individual clustering methods, as well as the ensemble methods including SAFE, SAME, and our CTEC methods, on the benchmark datasets in terms of ARI.

We also benchmarked our CTEC method for ensembling multiple clustering methods on the same five datasets. Figure 4 summarizes the performances of the five individual methods and the different ensemble methods, gauged by the ARI values. CTEC produced the best ensemble clustering results in four out of the five datasets, while achieving comparable results to the SAFE-clustering in the smallest dataset (Paul15). As for the comparison to the individual methods, the ensemble results produced by CTEC can further boost the best individual clustering results in four datasets, while keeping the high-quality clustering solution in the Pancreas dataset, in which the best individual method already produced good clustering solution with ARI of 0.76 (CIDR method), and the resulting ensemble still achieved ARI of 0.75 (CTEC-OB), compared to the ARI of 0.53 and 0.55 produced by the SAFE-clustering and SAME-clustering respectively. Although the authors of the SAME-clustering claimed that it can outperform the previous SAFE-clustering, SAME-clustering was worse than SAFE-clustering on ARI in four out of five datasets, which may be due to the effect of bad individual results, since the differences among the individual methods are much larger compared to the two-methods ensemble test. Compared to the other two state-of-the-art ensemble approaches, both versions of CTEC method produced better clustering ensemble on the five datasets on average. In general, CTEC-OB improved the ARI of the clustering results by 17.7% (to the SAFE-clustering) and 39.8% (to the SAME-clustering) on average across the five datasets, while the CTEC-DB improved the ARI of the clustering results for 19.9% (to the SAFE-clustering) and 42.4% (to the SAME-clustering) on average.

#### 3.2.1 Application to the cortex dataset

**Figure 5.**
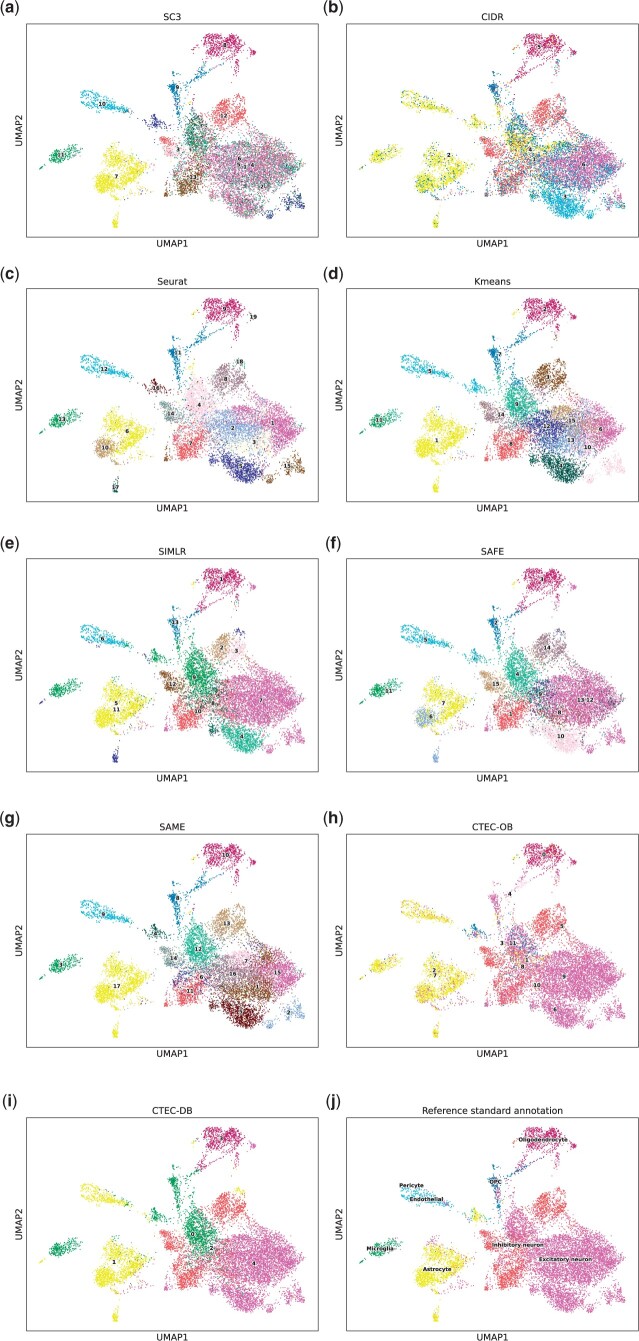
UMAP plots of the ensemble of five methods on the Cortex dataset. (a) SC3, (b) CIDR, (c) Seurat, (d) Kmeans, (e) SIMLR, (f) SAFE, (g) SAME, (h) CTEC-OB, (i) CTEC-DB, (j) Reference standard annotation. Compared with SAFE, SAME and other individual methods, CTEC yields relatively aggregative clustering results for excitatory neuron cells. In contrast, the clustering results are more diffused in the results of the other methods for excitatory neuron cells.

In this section, we take the Cortex dataset as an example to better understand the performances of our ensemble method. As shown in Fig. 4, the resulting ARI values of the five individual methods were low due to the unbalanced cell type distribution, three of which had even ARI values of lower than 0.2, posing challenges to the following ensemble process. The ARI values of the other two competing ensemble methods (i.e. 0.34 of SAFE and 0.18 of SAME) were lower than that of the best individual method (0.36 of SIMLR), suggesting that the ensemble results were highly affected by the clustering results with low quality. The resulting UMAP plots produced by the five individual methods and different ensemble methods on the Cortex dataset are shown in Fig. 5.

### 3.3 Ablation studies

#### 3.3.1 Impact of different base clustering methods

We evaluated the stability of our CTEC methods on Pancreas dataset by swapping the clustering method 1 and method 2 as input, and we tested all combinations for ensemble with a pair of methods from five methods. Table 1 shows that our method is not obviously affected by the input order of the two methods in most cases. In order to analyze the impact of different base clustering methods, the results in Table 1 show that the SIMLR method performs relatively worse when combined with all other base methods, which decreases the two methods ensemble performance (CTEC-OB ARI ranging from 0.50 to 0.69). However, when this SIMLR method is incorporated in the five-method ensemble, our CTEC-OB method achieves a more robust performance with ARI of 0.75 (Fig. 4).

**Table 1.** Performance evaluation of (a) CTEC-OB and (b) CTEC-DB (two methods ensemble) results in terms of ARI on the Pancreas dataset from all pairwise combinations of the seven individual clustering methods.

(a) ARI of CTEC-OB					
Base Methods	SC3	CIDR	Seurat	Kmeans	SIMILR
SC3		0.62	0.67	0.68	0.55
CIDR	0.73		0.78	0.78	0.69
Seurat	0.66	0.74		0.53	0.5
Kmeans	0.65	0.74	0.53		0.5
SIMILR	0.48	0.67	0.51	0.51	

(b) ARI of CTEC-DB					
Base Methods	SC3	CIDR	Seurat	Kmeans	SIMILR
SC3		0.62	0.68	0.68	0.51
CIDR	0.73		0.76	0.76	0.68
Seurat	0.67	0.79		0.53	0.51
Kmeans	0.67	0.78	0.53		0.5
SIMILR	0.66	0.71	0.51	0.51	

#### 3.3.2 Time and memory consumption

As shown in Table 2, we compared three recent single-cell clustering techniques [Secuer (Wei *et al.* 2022), SC3s (Quah and Hemberg 2022), and Scarf (Dhapola *et al.* 2022)] on five datasets with the CTEC (two methods ensemble from Leiden and DESC) in terms of ARI, NMI, time and memory consumption. The true number of clusters of the datasets are provided and the correspond best resolutions are calculated for each of the methods to ensure a fair comparison. Generally, CTEC outperforms these compared methods on the five benchmark datasets in terms of ARI and NMI. In terms of time consumption, both CTEC-DB and Scarf consume (0.34 s on average) about 70% less than other compared methods, and in the meanwhile CTEC-OB consumes 14.1 s on average, which is greater than Secuer and SC3s methods, and much less than DESC method. However, in terms of memory usage, the CTEC method used 15% more than Scarf and generally 70% fewer memories than other compared methods on the five datasets. All the computation was performed on the CPUs of 8 cores (AMD R7 3700X) and 48 GB RAMs. Overall, CTEC can integrate multiple clustering solutions and performs further refinements to produce better clustering ensemble while using less or similar memory and keeping shorter or similar running time (from CTEC-OB).

**Table 2.** Performance comparison of the Secuer, SC3s, Scarf, and CTEC methods (two methods ensemble from Leiden and DESC) on five benchmark datasets.

ARI					
Method	Benchmark datasets				
	Macaque	PBMC	Cortex	Pancreas	Paul15
Secuer	0.780	0.702	0.373	0.566	0.285
SC3s	0.930	0.763	0.433	0.643	0.324
Scarf	0.547	0.725	0.390	0.529	0.432
CTEC-OB	0.950	0.788	0.715	0.937	0.357
CTEC-DB	0.953	0.787	0.728	0.955	0.405

NMI					
Method	Benchmark datasets				
	Macaque	PBMC	Cortex	Pancreas	Paul15
Secuer	0.869	0.678	0.564	0.757	0.547
SC3s	0.923	0.713	0.551	0.791	0.535
Scarf	0.655	0.659	0.476	0.636	0.598
CTEC-OB	0.945	0.744	0.702	0.917	0.516
CTEC-DB	0.947	0.746	0.715	0.931	0.521

Time consumed (s)					
Method	benchmark datasets				
	Macaque	PBMC	Cortex	Pancreas	Paul15
Secuer	4.35	4.56	4.83	2.3	1.45
SC3s	3.72	2.57	0.87	1.09	0.72
Scarf	0.45	0.4	0.3	0.27	0.26
CTEC-OB	22.27	17.58	18.64	7.44	4.88
CTEC-DB	0.34	0.32	0.33	0.44	0.28

Memory using (MB)					
Method	benchmark datasets				
	Macaque	PBMC	Cortex	Pancreas	Paul15
Secuer	1081	897	2519	1985	361
SC3s	1072	881	2522	1984	347
Scarf	387	381	357	364	326
CTEC-OB	426	429	414	412	408
CTEC-DB	430	438	417	416	412

#### 3.3.3 Performance on large-scale dataset

A large-scale dataset named COVID-19 (GSE158055) cov [2021] was analyzed with our proposed method based on the two methods ensemble. This dataset contains 1 462 702 cells and 27 943 genes. We selected 1000 highly variable genes in pre-processing, and used its label ‘majorType’ (12 clusters) as the reference standard annotation. The computation was performed on the CPU of 64 cores [Intel(R) Xeon(R) Platinum 8255C CPU @ 2.50 GHz] and 350 G RAMs. The true number of clusters of the dataset has been provided to Scarf method, and the best resolutions have been calculated for Leiden and DESC methods to ensure a fair comparison. The Leiden method achieved a resolution of 0.0946044921875, while the DESC method had a resolution of 0.115966796875. As can be seen in Table 3, CTEC-OB performed similarly to the Scarf method and outperformed the DESC methods in terms of ARI and NMI. The Leiden method had the best performance overall. However, the DESC method did not achieve an optimistic result, which also affected the performance of CTEC-DB. One possible reason for this could be that the complexity of the neural network in DESC may not be well adapted to the scale of the COVID-19 dataset. Moreover, the Scarf used only 59.1 Gigabytes of memory and short end-to-end time consuming (less than 10 min) on this large-scale dataset based on the zarr format, while Leiden and DESC used more than 200 Gigabytes of memory. Generally, CTEC can be readily scaled up and applied to the larger scRNA-seq datasets.

**Table 3.** Performance evaluation of CTEC methods on large-scale dataset COVID-19.

Method	ARI	NMI	Time consuming (s)
Leiden	0.665	0.741	3430.41
DESC	0.555	0.584	23845.2
CTEC-OB	0.602	0.691	5198.83
CTEC-DB	0.532	0.660	6.83
Scarf	0.612	0.669	35.1

Two more ablation studies provided further insights into the contribution of several strategies of the proposed method: (i) In the ensemble clustering of two individual methods, the contribution of iterative cluster labels updating was evaluated on both outlier- and distribution-based and re-clustering strategies, leading to an improvement of 12.9% (outlier-based) and 14.9% (distribution-based) in terms of ARI, respectively. (ii) For the multiple methods-based ensemble, the cluster quality evaluation with bad cluster removal based on the unsupervised CH score was further evaluated. As a result, there was an improvement of 28.5% (outlier-based) and 26.8% (distribution-based) in terms of ARI with this process. The code and the reproducible scripts are publicly available on https://github.com/LWCHN/CTEC.git. In addition, the five benchmarking datasets in the h5ad format are also available on google drive link.

## 4 Discussion

In this study, we have proposed an ensemble clustering method, termed CTEC, for scRNA-Seq data clustering. Without the prior knowledge of cell-type proportion and optimized parameters of each dataset, we found that the individual methods did not yield the best clustering results based on their default parameters. However, the proposed CTEC method improved the clustering results based on the cross-tabulation and iterative subgroup cells refinements on five benchmark datasets.

The CTEC method integrates two clustering results with the cross-tabulation ensemble to produce consensus clustering results with high quality, and there are two re-clustering strategies (distribution-based and outlier-based re-clustering) that can be performed depending on the properties of the dataset and the individual results that need to be integrated. According to the results, the distribution-based re-clustering performed better than the outlier-based version for the two-methods (Leiden and DESC) ensemble tests, while slightly worse (or similar) in three out of the five datasets when ensembling multiple methods. A reasonable explanation could be that most of the consensus clustering results between Leiden and DESC were correct. The distribution-based re-clustering trusted all of the consensus clustering results when conducted re-clustering, while the outlier-based re-clustering assumed that there were several wrongly clustered cells in the consensus clustering results which should be excluded by the outlier detection method. In our experiments, the outlier detection method may have excluded a few correctly clustered cells during outlier-based re-clustering, which led to worse clustering performances than distribution-based re-clustering. However, outlier-based re-clustering provides the potential to deal with consensus clustering that contains a few wrong consensus results from the individual methods. One of the limitations in our study is the lacking of generated private data to conduct biologically meaningful analysis, which could be addressed in future research.

In conclusion, CTEC can be considered as a parameter-free single-cell clustering method to improve the individual methods without any prior knowledge of cell type and parameter optimization information, which has potential to be exploited as a valuable tool by biomedical researchers to facilitate their single-cell analysis. Moreover, in combination with the advantages of integrating multiple clustering methods and shorter computing time, CTEC may serve as a plug-in in cases where users have difficulties finding the most suitable clustering methods or parameter settings for their data to provide ensemble solutions with high quality, which can benefit the solution of downstream tasks.

## Supplementary Material

btae130_Supplementary_Data
