# Supplementary material for: CTEC: a cross-tabulation ensemble clustering approach for single-cell RNA sequencing data analysis
Source: Bioinformatics. 2024 Mar 29;40(4):btae130. doi: 10.1093/bioinformatics/btae130 (PMC10985676; doi:10.1093/bioinformatics/btae130)
Supplement: btae130_Supplementary_Data [file btae130_supplementary_data.pdf]

*Bioinformatics*, 2024, pp. 1–8

**doi: 10.1093/bioinformatics/xxxxx**
Advance Access Publication Date: Day Month Year
Paper

PAPER

# Supplementary materials: CTEC: a cross-tabulation ensemble clustering approach for single-cell RNA sequencing data analysis

Liang Wang,[1,†] Chenyang Hong,[2,†] Jiangning Song[3] and Jianhua Yao[1,*]

[1]AI Lab, Tencent, No. 33, Haitian Second Road, 518054, Shenzhen, China, [2]Department of Computer Science and Engineering, The Chinese University of Hong Kong, New Territories, Hong Kong, China and [3]Biomedicine Discovery Institute and Department of Biochemistry and Molecular Biology, Monash University, 3800, VIC, Australia

[†]These authors made equal contributions.[*]Corresponding author. jianhuayao@tencent.com

FOR PUBLISHER ONLY Received on Date Month Year; revised on Date Month Year; accepted on Date Month Year

## Abstract

## Benchmarking datasets details

We collected five benchmarking datasets that have been commonly used for performance evaluation of recent works in the literature Li et al. [2020], Lakkis et al. [2021]. A statistical summary of these datasets is described in **Table 1**.

## Ensemble with two methods

### Application to Macaque dataset

The ARIs of the proposed CTEC methods on the Macaque dataset were 0.91 to 0.95, and the NMIs were 0.92 to 0.94, respectively. The results based on the setup of distribution-based re-clustering were generally better than that of outlier-based re-clustering. All of these results were better than Leiden (ARI: 0.79, NMI: 0.87) and DESC results (ARI: 0.88, NMI: 0.92). The SAFE-clustering method obtained slightly worse ARI (0.76) and NMI (0.84) when compared with the Leiden and DESC results, while the SAME-clustering also generated a better ensemble compared with the individual methods, with ARI of 0.93 and NMI of 0.94, but still slightly worse than the CTEC results.

The clustering results of all the methods can be found in **Fig. S1** in the form of the UMAP plot. For the cluster #6 of CTEC-DB in **Fig. S1[f]**, we found that the Leiden clusters #3, #10, #13 and other clusters in **Fig. S1[a]** were re-grouped together, the reference standard annotation in **Fig. S1[f]** also supports this grouping, since this cluster corresponds to the RB cells. However, **Fig. S1[c]** shows that the RB cells were divided into three clusters (#4, #5, #6) by the SAFE method, which yielded less clustering accuracy, while the SAME method also did not group them into one cluster in **Fig. S1[d]**. From **Fig. S1[a]** of the Leiden results, the three clusters (#0, #15, #17) on the middle left of the plot, were merged to one cluster (#0) in the CTEC-DB results in **Fig. S1[f]**. This re-clustering was consistent to the reference standard annotation of cell annotation IMB in **Fig. S1[f]**. In addition, although the RB cells in CTEC-OB results in **Fig. S1[e]** were represented by four clusters (#2, #5, #14,#15), most of the cells were represented by the same color as RB cells from **Fig. S1[g]**. It means that three of the four clusters (#2, #5, #14,#15) could be considered as outlier cell clusters.

### Application to Pancreas dataset

This data was generated from four public datasets by using four protocols of CEL-seq Grün et al. [2016], CEL-seq2 Muraro et al. [2016], Fluidigm C1 Lawlor et al. [2017], and SMART-seq2 Segerstolpe et al. [2016]. With the optimized parameters ($resolution\_use = 0.2$) in `https://eleozzr.github.io/desc/reproduce.html`, the DESC declared a clustering result with the ARI of 0.95. In our implementation, we obtained ARI of 0.60 and NMI of 0.80 from the DESC method using its default parameters ($resolution = 1.0$), which were better than those of the Leiden method with the performance of 0.43 (ARI) and 0.74 (NMI). The SAFE method performed similarly to DESC with the accuracies of 0.50 (ARI) and 0.74 (NMI). The SAME clustering outperformed the individual results with the ARI of 0.63 and NMI of 0.81. The ensemble clustering result produced by CTEC-DB was improved by 12.8% in terms of ARI than the DESC result, which was 0.68. Therefore, CTEC shown a good ability to extract the consensus information from the individual clustering results correctly which made better clustering accuracy. **Fig. S2** shows the UMAP plot for these methods. The alpha cells shown in **Fig. S2**(g) were clustered into four clusters (#1, #3, #4, #15) by DESC (**Fig. S2**(b)), and seven clusters (#2, #3, #6, #9, #11, #15, #19) by Leiden (**Fig. S2**(a)). The CTEC-DB in **Fig. S2**(f) combined these clusters into three clusters (#7,#8,#12). Considering the alpha cells from the reference standard annotation, we

© The Author 2024. Published by Oxford University Press. All rights reserved. For permissions, please e-mail: journals.permissions@oup.com

found that the Leiden and DESC results show different clusters distributions separately. The CTEC-DB method enabled these different clustering distributions to complement each other with the correct cluster information. In addition, the SAFE method (**Fig. S2**(c)) yielded four clusters (#2, #3, #4, #5) on the alpha cells, and the SAME method (**Fig. S2**(d)) reduced them into three clusters (#2, #6, #12) which was slightly better than Leiden and DESC results but was still worse than the CTEC ensemble results.

## Application to PBMC and Paul15 datasets

The UMAP plots for the PBMC dataset are shown in **Fig. S3**, while the plots for the Paul15 dataset are shown in **Fig. S4**.

## Comparisons of NMI for the benchmark datasets based on two methods ensemble

The NMI values of the clustering results generated by the two individual methods (Leiden and DESC) as well as their consensus clustering results from different ensemble methods are shown in **Fig. S5**.

# Ensemble with SAFE and SAME method with four or five input methods

We evaluated the ensemble results of the SAFE and SAME methods by taking four or five individual methods as the input. For the input with four methods, we removed the individual clustering result with the lowest Calinski-Harabasz score as in the CTEC method. The evaluation of ARI values is shown in **Fig. S6**. For the SAFE method, the ensemble results with all five methods outperform the four-method results on three of the five datasets, while for the SAME method the five-method ensemble achieved better results in four datasets. Overall, the ensemble quality with four or five methods is similar to each other.

### *CTEC ensemble with five methods*

The resulting UMAP plots generated by the five individual methods and different ensemble methods of the Macaque dataset are shown in **Fig. S7**. The plots for the Pancreas dataset are shown in **Fig. S9**. The plots for the PBMC are shown in **Fig. S8**. The plots for the Paul15 dataset are shown in **Fig. S10**.

As shown in Table 2, The computing time on the Macaque dataset (30,302 cells) were 193.8, 28882.2, 112.8, and 0.6 seconds for the SAFE-clustering, SAME-clustering, CTEC-OB, and CTEC-DB methods, respectively. based on the CPU of 10 cores (Intel(R) Xeon(R) Platinum 8255C CPU @ 2.50GHz) and 40 G RAMs. Even on a smaller dataset of Pancreas with 6,321 cells, SAME-clustering took 1642.2 seconds on clustering compared to the shorter running time of 10.2, 16.2, and 0.3 seconds for SAFE-clustering, CTEC-OB, and CTEC-DB methods.

## Ablation studies

The cluster quality evaluation is applied based on the unsupervised Calinski-Harabasz (CH) score Caliński and Harabasz [1974], which is a metric for clustering evaluation without the ground truth labels and is highly correlated with the ground-truth ARI value **Fig. S11**.

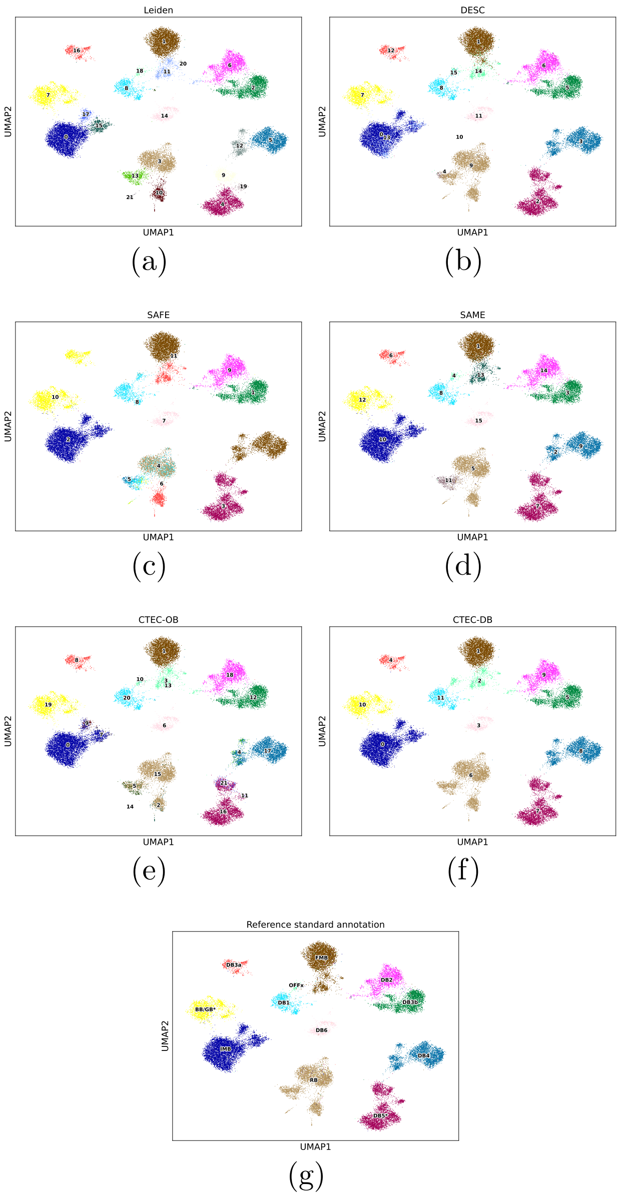


**Fig. S1.** UMAP plots of all methods for the Macaque dataset.

**Table 1.** Statistical summary of the datasets used in this study. The true clusters refer to the cell annotation of the original work. Paul15 refers to the mouse bone marrow dataset by Paul in 2015.

| Dataset name | Species and tissue | Number of cells | Number of True clusters | Ref |
|---|---|---|---|---|
| Macaque | Macaque retina | 30,302 | 12 | GSE118480 Peng et al. [2019] |
| PBMC | Human PBMC | 24,679 | 8 | GSE96583 Kang et al. [2018] |
| Cortex | Mouse cortex | 13,783 | 8 | SCP425 Ding et al. [2020] |
| Pancreas | Human pancreas | 6,321 | 13 | GSE81076 Grün et al. [2016]<br>GSE85241 Muraro et al. [2016]<br>GSE86469 Lawlor et al. [2017]<br>E-MTAB-5061 Segerstolpe et al. [2016] |
| Paul15 | Mouse bone marrow | 2,730 | 10 | GSE727857 Paul et al. [2015] |

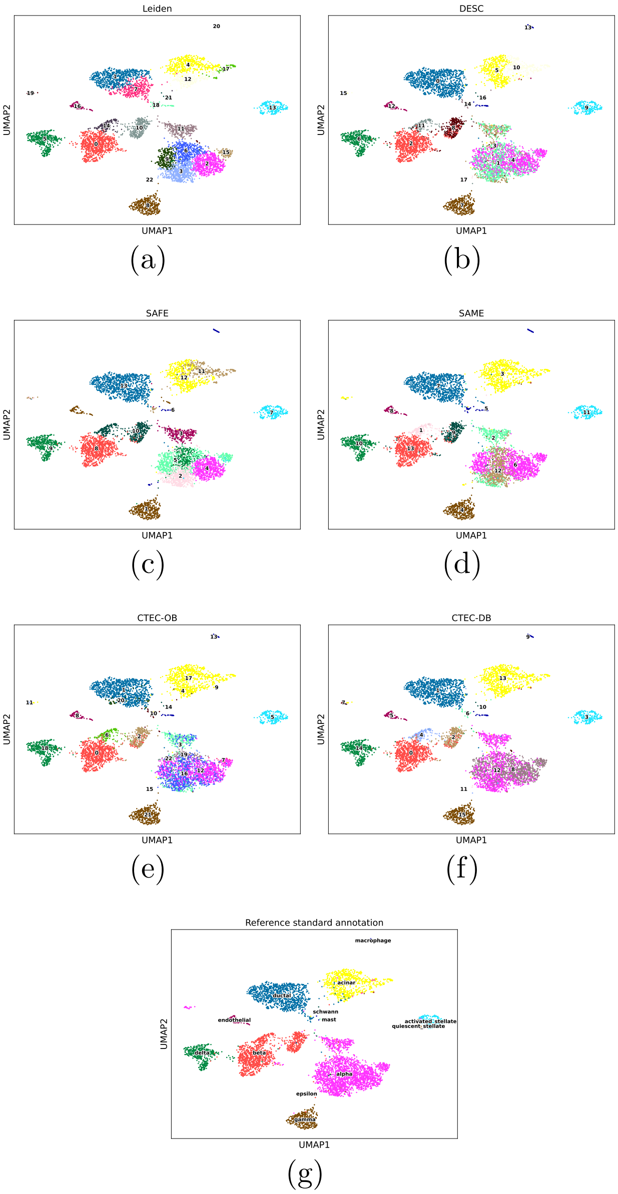


**Fig. S2.** UMAP plots of the methods for the Pancreas dataset.

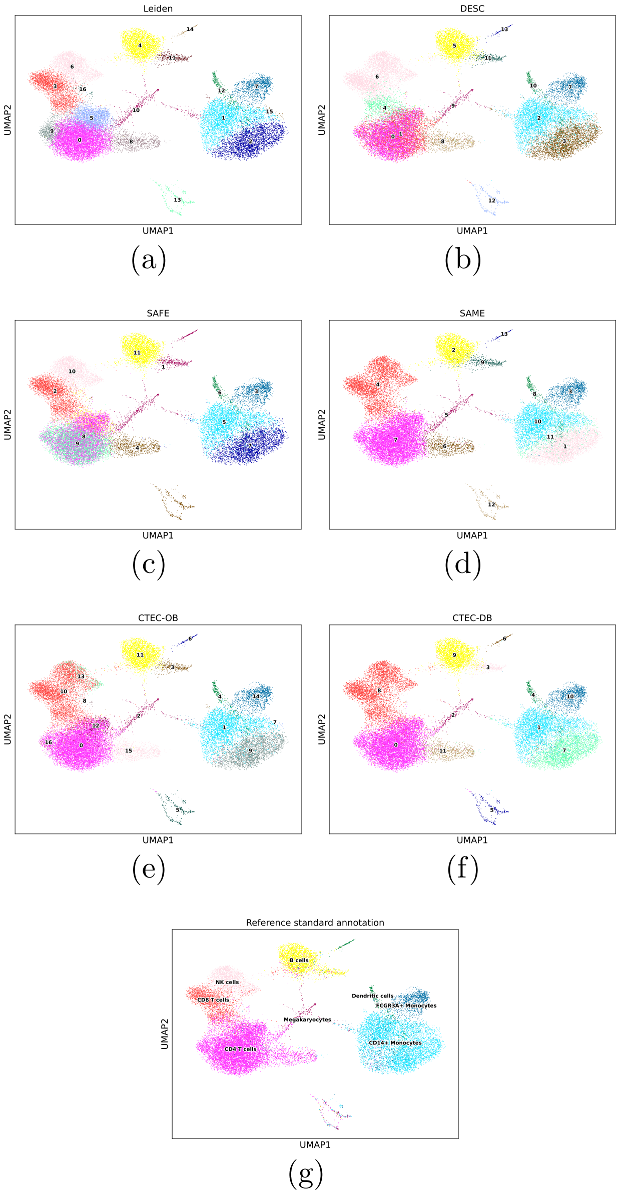


**Fig. S3.** UMAP plots of the methods for the PBMC dataset.

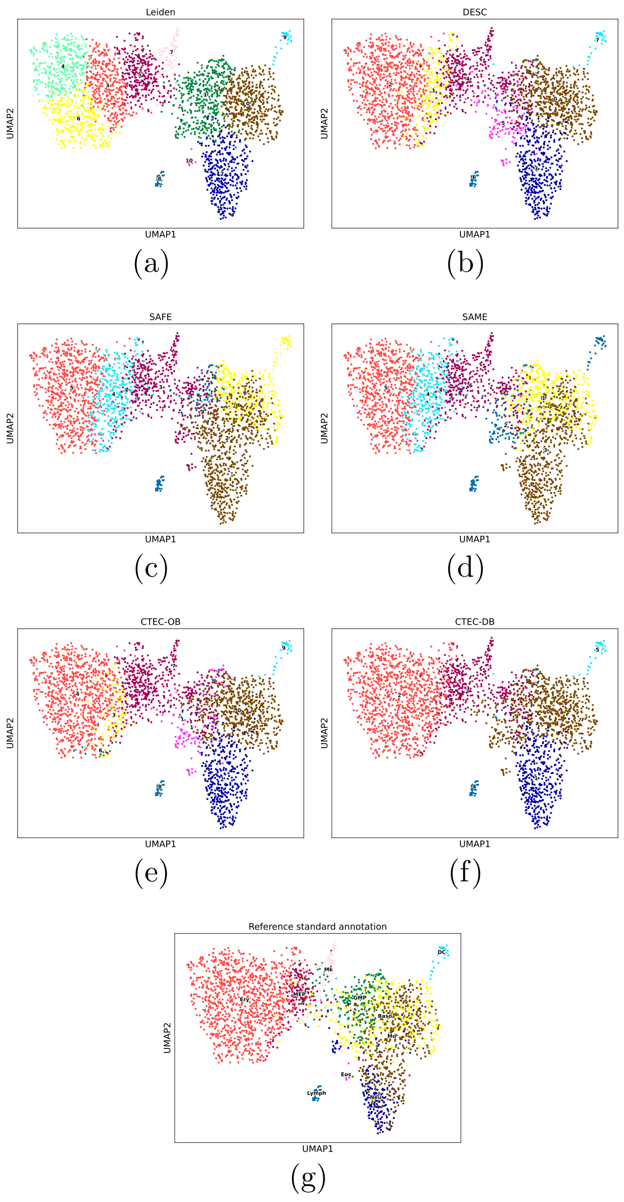


**Fig. S4.** UMAP plots of the methods for the Paul15 data.

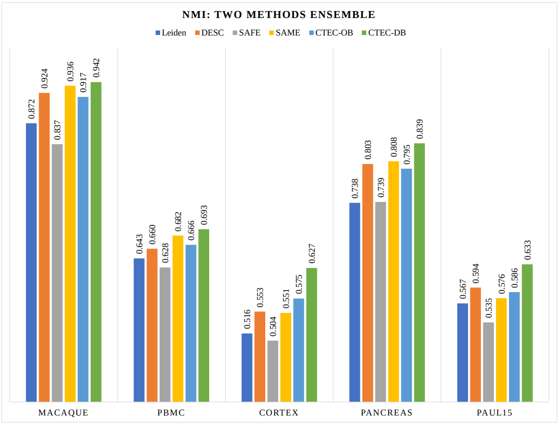


**Fig. S5.** Performance comparisons of the SAFE, SAME, and CTEC methods based on two methods ensemble (Leiden and DESC) in terms of NMI on the benchmark datasets.

**Table 2.** Computing time comparison of the SAFE, SAME, and CTEC methods on two datasets.

| Dataset name | Macaque | Pancreas |
|---|---|---|
| SAFE-clustering | 193.8 secs | 10.2 secs |
| SAME-clustering | 28882.2 secs | 1642.2 secs |
| CTEC-OB | 112.8 secs | 16.2 secs |
| CTEC-DB | 0.6 secs | 0.3 secs |

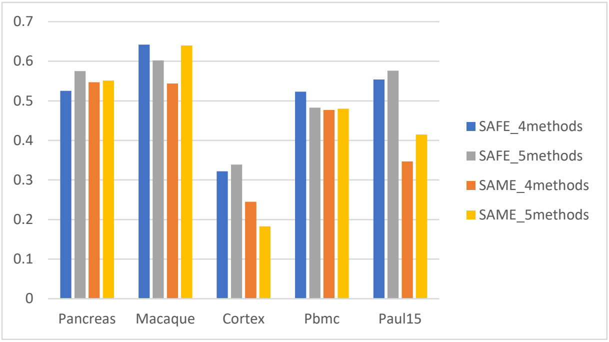


**Fig. S6.** Performance evaluation of the SAFE and SAME methods with two input settings in terms of ARI.

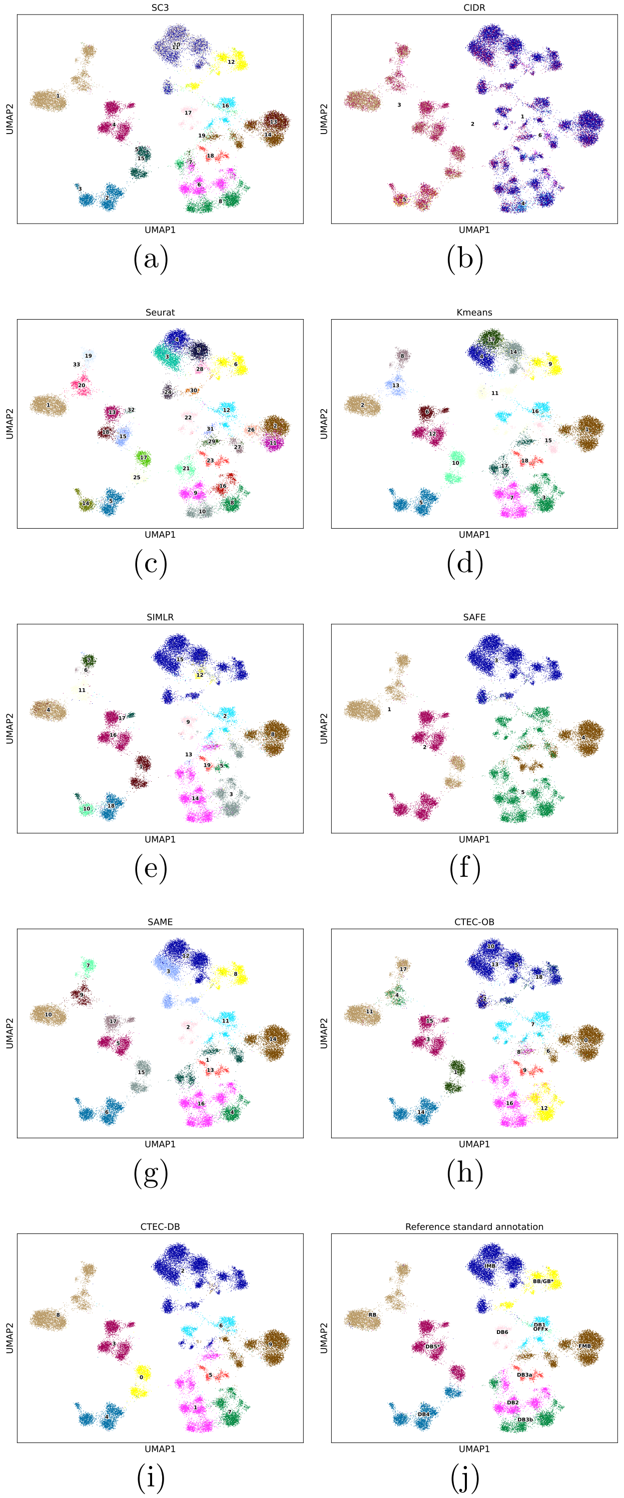


**Fig. S7.** UMAP plots of all methods for the Macaque data for five-method ensemble.

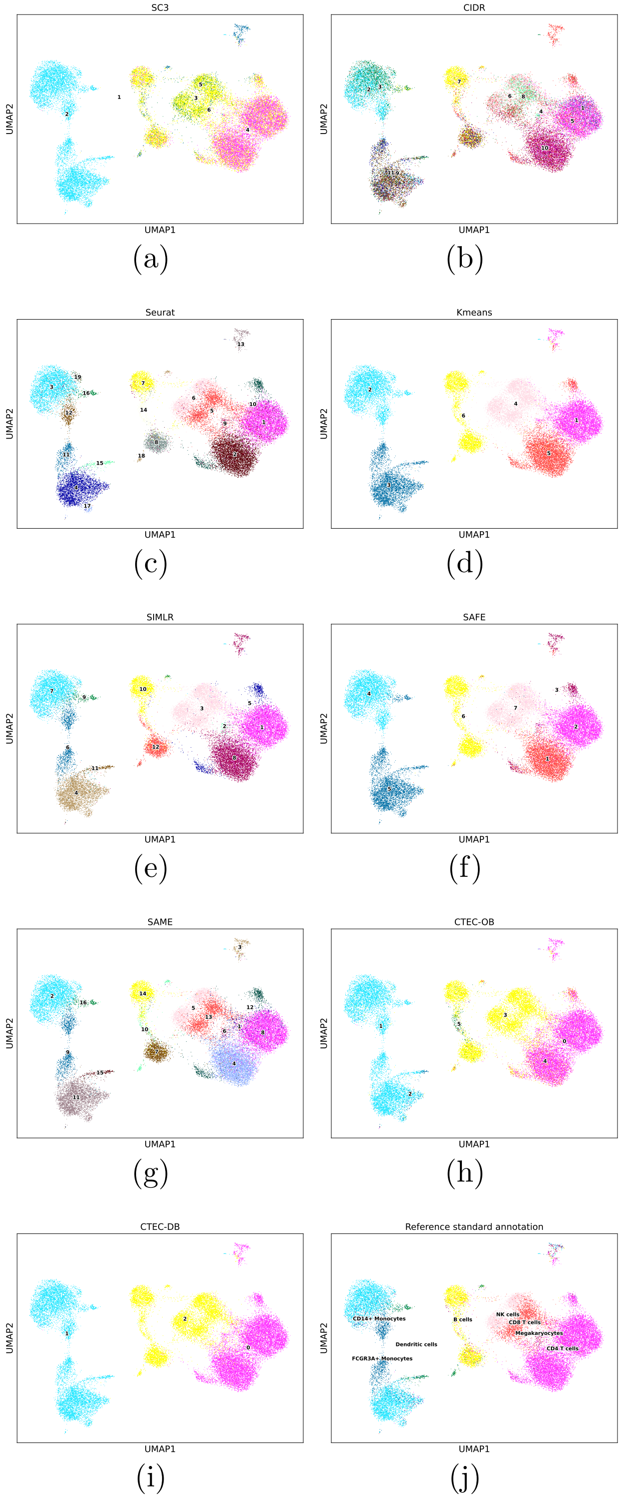


**Fig. S8.** UMAP plots of all methods for the PBMC data for five-method ensemble.

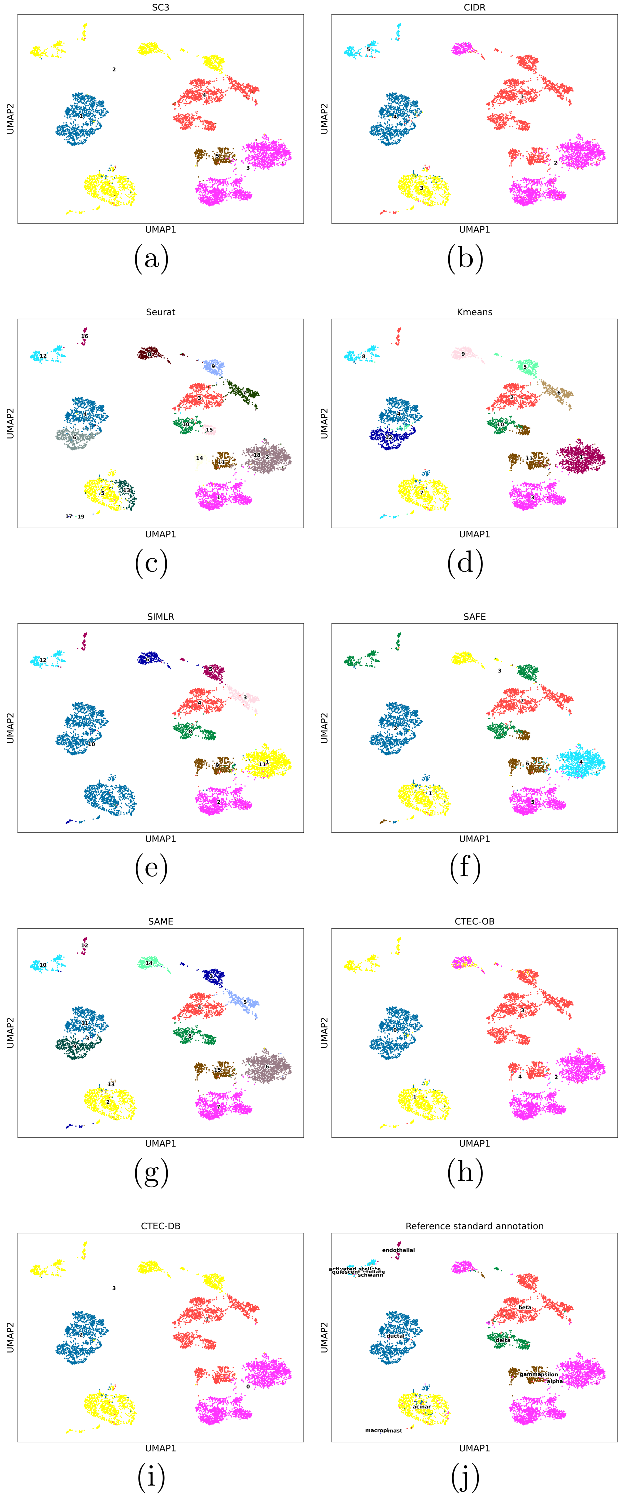


**Fig. S9.** UMAP plots of all methods for the Pancreas data for five-method ensemble.

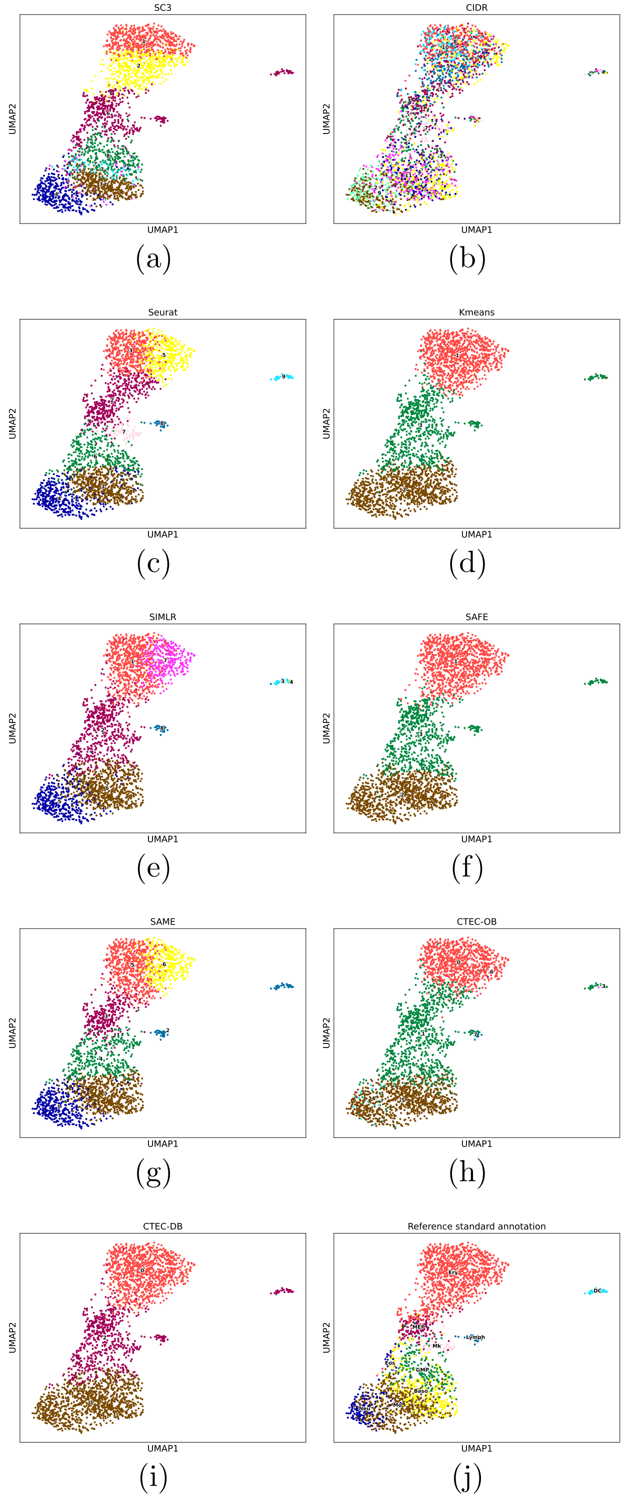


**Fig. S10.** UMAP plots of all methods for the Paul15 data for five-method ensemble.

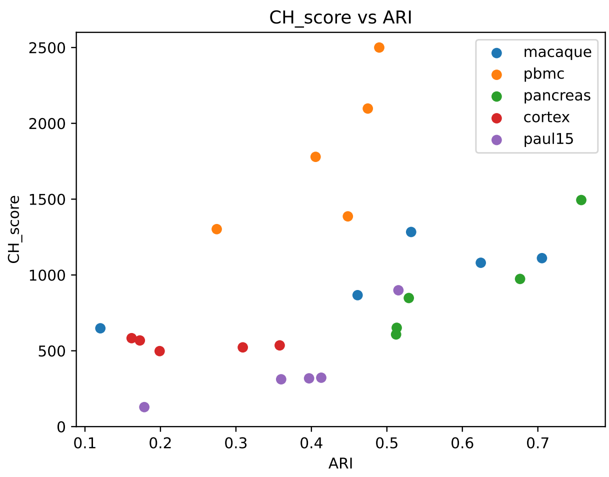


**Fig. S11.** Comparisions of Calinski-Harabasz score and ground-truth ARI for the five methods on the five datasets.

## References

T. Caliński and J. Harabasz. A dendrite method for cluster analysis. *Communications in Statistics-theory and Methods*, 3(1):1–27, 1974.

J. Ding, X. Adiconis, S. K. Simmons, M. S. Kowalczyk, C. C. Hession, N. D. Marjanovic, T. K. Hughes, M. H. Wadsworth, T. Burks, L. T. Nguyen, J. Y. H. Kwon, B. Barak, W. Ge, A. J. Kedaigle, S. Carroll, S. Li, N. Hacohen, O. Rozenblatt-Rosen, A. K. Shalek, A.-C. Villani, A. Regev, and J. Z. Levin. Systematic comparison of single-cell and single-nucleus RNA-sequencing methods. *Nature Biotechnology*, 38(6):737–746, jun 2020. ISSN 1087-0156. doi: 10.1038/s41587-020-0465-8.

D. Grün, M. J. Muraro, J.-C. Boisset, K. Wiebrands, A. Lyubimova, G. Dharmadhikari, M. van den Born, J. van Es, E. Jansen, H. Clevers, E. J. de Koning, and A. van Oudenaarden. De Novo Prediction of Stem Cell Identity using Single-Cell Transcriptome Data. *Cell Stem Cell*, 19 (2):266–277, aug 2016. ISSN 19345909. doi: 10.1016/j.stem.2016.05.010.

H. M. Kang, M. Subramaniam, S. Targ, M. Nguyen, L. Maliskova, E. McCarthy, E. Wan, S. Wong, L. Byrnes, C. M. Lanata, R. E. Gate, S. Mostafavi, A. Marson, N. Zaitlen, L. A. Criswell, and C. J. Ye. Multiplexed droplet single-cell RNA-sequencing using natural genetic variation. *Nature Biotechnology*, 36(1):89–94, jan 2018. ISSN 1087-0156. doi: 10.1038/nbt.4042.

J. Lakkis, D. Wang, Y. Zhang, G. Hu, K. Wang, H. Pan, L. Ungar, M. Reilly, X. Li, and M. Li. A joint deep learning model enables simultaneous batch effect correction, denoising and clustering in single-cell transcriptomics. *Genome Research*, page gr.271874.120, 2021. ISSN 1088-9051. doi: 10.1101/gr.271874.120.

N. Lawlor, J. George, M. Bolisetty, R. Kursawe, L. Sun, V. Sivakamasundari, I. Kycia, P. Robson, and M. L. Stitzel. Single-cell transcriptomes identify human islet cell signatures and reveal cell-type–specific expression changes in type 2 diabetes. *Genome Research*, 27(2):208–222, feb 2017. ISSN 1088-9051. doi: 10.1101/gr.212720.116.

X. Li, K. Wang, Y. Lyu, H. Pan, J. Zhang, D. Stambolian, K. Susztak, M. P. Reilly, G. Hu, and M. Li. Deep learning enables accurate clustering with batch effect removal in single-cell rna-seq analysis. *Nature communications*, 11(1): 1–14, 2020.

M. J. Muraro, G. Dharmadhikari, D. Grün, N. Groen, T. Dielen, E. Jansen, L. van Gurp, M. A. Engelse, F. Carlotti, E. J. de Koning, and A. van Oudenaarden. A Single-Cell Transcriptome Atlas of the Human Pancreas. *Cell Systems*, 3(4):385–394.e3, oct 2016. ISSN 24054712. doi: 10.1016/j.cels.2016.09.002.

F. Paul, Y. Arkin, A. Giladi, D. A. Jaitin, E. Kenigsberg, H. Keren-Shaul, D. Winter, D. Lara-Astiaso, M. Gury, A. Weiner, et al. Transcriptional heterogeneity and lineage commitment in myeloid progenitors. *Cell*, 163(7):1663–1677, 2015.

Y.-R. Peng, K. Shekhar, W. Yan, D. Herrmann, A. Sappington, G. S. Bryman, T. van Zyl, M. T. H. Do, A. Regev, and J. R. Sanes. Molecular Classification and Comparative Taxonomics of Foveal and Peripheral Cells in Primate Retina. *Cell*, 176(5):1222–1237.e22, feb 2019. ISSN 00928674. doi: 10.1016/j.cell.2019.01.004.

Å. Segerstolpe, A. Palasantza, P. Eliasson, E.-M. Andersson, A.-C. Andréasson, X. Sun, S. Picelli, A. Sabirsh, M. Clausen, M. K. Bjursell, D. M. Smith, M. Kasper, C. Ämmälä, and R. Sandberg. Single-Cell Transcriptome Profiling of Human Pancreatic Islets in Health and Type 2 Diabetes. *Cell Metabolism*, 24(4):593–607, oct 2016. ISSN 15504131. doi: 10.1016/j.cmet.2016.08.020.
